# Metagenomic analysis reveals the different characteristics of microbial communities inside and outside the karst tiankeng

**DOI:** 10.1186/s12866-022-02513-1

**Published:** 2022-04-26

**Authors:** Cong Jiang, Xiao-Rui Sun, Jie Feng, Su-Feng Zhu, Wei Shui

**Affiliations:** 1 College of Urban and Environmental Sciences, Peking University, Beijing, 100871 China; 2 College of Environment Safety Engineering, Fuzhou University, Fuzhou, 350116 China; 3 Chinese Research Academy of Environmental Sciences, Beijing, 100020 China

**Keywords:** Karst tiankeng, Biodiversity, Metagenome, Microbial function

## Abstract

**Background:**

Karst tiankengs serve as a reservoir of biodiversity in the degraded karst landscape areas. However, the microbial diversity of karst tiankengs is poorly understood. Here, we investigated the composition and function of the microbial community in a karst tiankeng.

**Results:**

We found that habitat differences inside and outside the karst tiankeng changed the composition and structure of the soil microbial communities, and the dominant phyla were *Proteobacteria*, *Actinobacteria*, *Chloroflexi* and *Acidobacteria.* The Shannon–Wiener diversity of microbial communities inside and outside the tiankeng was significantly different, and it was higher inside the tiankeng (IT). Venn and LEfSe analysis found that the soil microbial communities inside the tiankeng had 640 more endemic species and 39 more biomarker microbial clades than those identified outside of the tiankeng (OT)..Functional prediction indicated that soil microorganisms in outside the tiankeng had a high potential for carbohydrate metabolism, translation and amino acid metabolism. There were biomarker pathways associated with several of human diseases at both IT and OT sites. Except for auxiliary activities (AA), other CAZy classes had higher abundance at IT sites, which can readily convert litter and fix carbon and nitrogen, thereby supporting the development of underground forests. The differences in microbial communities were mainly related to the soil water content and soil total nitrogen.

**Conclusions:**

Our results provide a metagenomic overview of the karst tiankeng system and provide new insights into habitat conservation and biodiversity restoration in the area.

**Supplementary Information:**

The online version contains supplementary material available at 10.1186/s12866-022-02513-1.

## Introduction

Population growth, overexploitation of land and climate change have severely affected many ecosystems on Earth. The most immediate consequences are the loss of biodiversity and changes in ecosystem services [1]. Biodiversity plays crucial roles in ecosystem functions (e.g., nutrient cycling rates, stability and productivity) [2, 3]. Karst landscape area are characterized by significant soil erosion, sparse vegetation and extensive exposure of bedrock and are considered to be one of the most eroded regions in the world [4]. However, the karst tiankeng isa different kind of area. Karst tiankengs, as a new kind of grand negative phenomenon, were first characterized in 2001 [5]. Due to the sides of the surrounding rock walls, the interior of the karst tiankeng has a unique climate and hydrothermal conditions that are different from those outside the tiankeng, and this area has bred rich and unique animal, plant and microbial resources [6, 7]. As an oasis of degraded karst landscapes, karst tiankengs may become an important habitat and conservation reservoir for certain species under global climate change [8, 9]. China is the “world karst tiankeng kingdom”; of the more than 200 karst tiankengs that have been discovered worldwide, approximately 170 are located in China, with a large number of typical karst tiankeng groups [10]. The development of tourism and cave exploration promoted the discovery and research of karst tiankengs.

In recent decades, research on karst tiankengs has mainly focused on geology [10], plant communities [5, 11] and organic pollutants [12]. Especially in the study of plant composition and structure, the area inside tiankengs possesses higher plant diversity and more unique flora than areas outside these environments. For example, remarkably higher diversity at all plant taxonomic levels was found inside the Chuandong tiankeng, demonstrating the ancient and isolated nature of the tiankeng flora [5]. Our vegetation survey of the Shengxiantang tiankeng also found that, compared with the habitat outside the tiankeng, the trees and shrubs inside the tiankeng had higher species richness and species diversity (Unpublished). In addition, existing research has shown that a relatively closed and unique microclimate environment with stable temperature, abundant rainfall and suitable humidity is formed inside the tiankeng [10]. The soil inside the tiankeng has higher moisture, nutrients and litter than soil outside. The establishment of an ecosystem is a long-term process, and the intricacy of the interactions between aboveground and underground biological communities promotes the diversity of the ecosystem and determines the development of biological communities [13, 14]. Soil microorganisms are crucial components of the terrestrial biosphere and participate in biogeochemical processes, such as the C and N cycles [15, 16]. Previous studies have found that soil nutrient status and vegetation have a pronounced influence on microbial community structure and activities in karst ecosystems [17, 18]. Therefore, it is reasonable to speculate that the different climatic conditions, soil environment and vegetation cover inside and outside the tiankeng may affect the structure and function of microbial communities. Studies investigating the characteristics of the microbial community in karst tiankengs are important for ecological restoration and biodiversity protection. However, the understanding of karst tiankeng soil microbial community diversity is still incomplete. This knowledge gap limits our understanding of the impact of biodiversity changes on ecosystem function and the value of the karst tiankeng biodiversity conservation library in terms of climate change.

By using metagenomic sequencing in this study, we explored the characteristics of the microbial community in Shenxiantang tiankeng which belongs to the Zhanyi tiankeng group in Yunnan, China. Here, we explored whether the unique karst tiankeng habitat has changed the composition, structure, and function of the soil microbial community from outside to inside by examining soil samples from both inside and outside of the tiankeng. Thus, we hypothesized that (i) soil microbial community diversity and composition differ between the inside and outside the tiankeng; (ii) the differentiated environment inside and outside the tiankeng alters the function of the soil microbial community; and (iii) changes in the soil microbial community are largely due to variations in soil physiochemical properties.

## Results

### Soil physiochemical properties and plant cover

Compared with the outside of the tiankeng, the soil physiochemical properties inside the tiankeng has underwent obvious changes. Almost all soil physiochemical properties inside the tiankeng (IT) were higher than those outside the tiankeng (OT), except for total phosphorus (TP). The total nitrogen (TN) and soil water content (SWC) were significantly higher at the IT sites (*P* < 0.05). The soil samples were all slightly acidic, with a pH value between 6.62 and 6.50, and the OT was lower than the IT (*P* > 0.05) (Table 1). The tiankeng habitat also changed the plant characteristics. The plant cover was highest at the IT sites, and the Shannon–Wiener index was 2.28 (Table S1).

**Table 1:** Soil physicochemical properties of different Shenxiantang tiankeng sites

Site	TOC (g/kg)	TN (g/kg)	TP(g/kg)	TK(g/kg)	pH	SWC (%)
IT	42.07 ± 3.57a	3.53 ± 0.73a	0.66 ± 0.12a	18.01 ± 2.66a	6.62 ± 0.21a	47.40 ± 3.12a
OT	40.40 ± 2.62a	2.65 ± 0.74b	0.67 ± 0.08a	15.39 ± 5.19a	6.50 ± 0.27a	43.07 ± 4.37b

Different minuscule alphabet indicate divergence is significant (*P* < 0.05)

### Soil microbial structures and composition

Approximately 818 million sequences were obtained from 15 samples, averaging approximately 55 million per sample. Among all sequences, 92.47% passed quality control for downstream analysis. The annotated metagenomic sequences were most often assigned into bacteria (99.30% ~ 98.91%), archaea (0.75% ~ 0.47%) and fungi (0.20% ~ 0.30%) (Table S2). There were also a few annotated metagenomic sequences assigned to viruses in both soil samples. The Shannon–Wiener index was significantly higher at the IT sites (13.55) than at the OT sites (13.14) (*P* < 0.05) (Fig. S1).

**Fig. 1:**
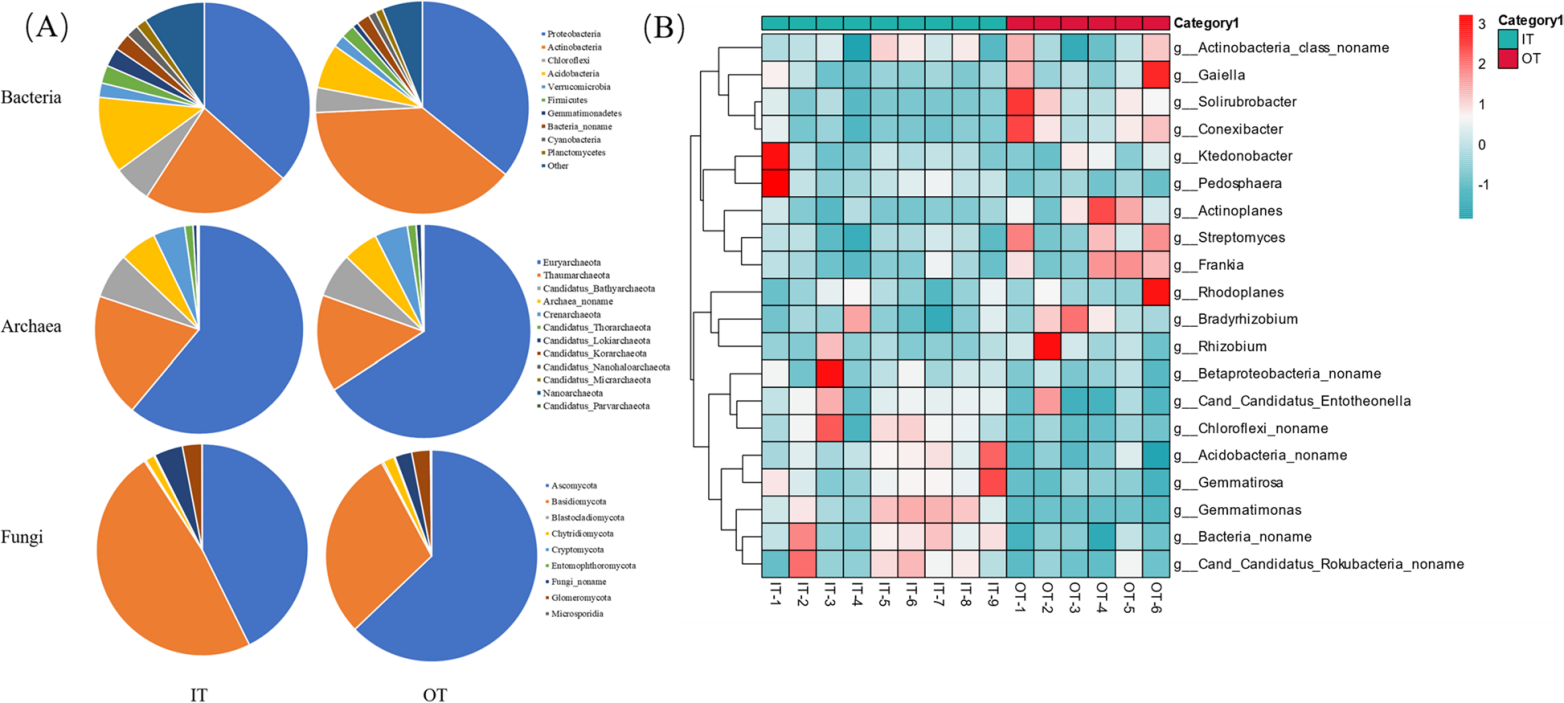
The microbial community composition of different Shenxiantang tiankeng sites at the phyla level (**A**) and at the genus level (top 20) (**B**)

A total of 106 phyla were detected in the Shenxiantang tiankeng and were present in all soil samples, including 84 bacteria, 12 archaea, 9 fungi and 1 virus. Microbial communities showed differences in abundance at the phylum classification level. As shown in Fig. 1A, the most abundant bacterial phyla were *Proteobacteria*, *Actinobacteria*, *Chloroflexi* and *Acidobacteria.* Among them, *Proteobacteria* was more abundant in the IT than in the OT, and *Actinobacteria* was more abundant in OT than in the IT. The most abundant archaeal phyla were *Euryarchaeota, Thaumarchaeota,* and *Candidatus_Bathyarchaeota.* As an important group of soil microbial communities, the most abundant fungal phyla were *Ascomycota* and *Basidiomycota*. At the genus level (Fig. 1B), the top 20 classes were screened. The most abundant genus of microbial communities was *Bradyrhizobium*, followed by *Betaproteobacteria_noname*, *Streptomyces* and *Solirubrobacter*. Furthermore, the abundances of *Bacteria_noname*, *Ktedonobacter* and *Acidobacteria_noname* varied among the different sites. For example, *Bradyrhizobium* had a higher relative abundance in the OT, as was *Betaproteobacteria_noname* in the IT and *Streptomyces* in the IT. Furthermore, the Venn diagram revealed 16,472 taxa of microbial communities, and the site-specific taxonomy ranged from 985 (IT) to 345 (OT) (Fig. S2).

**Fig. 2:**
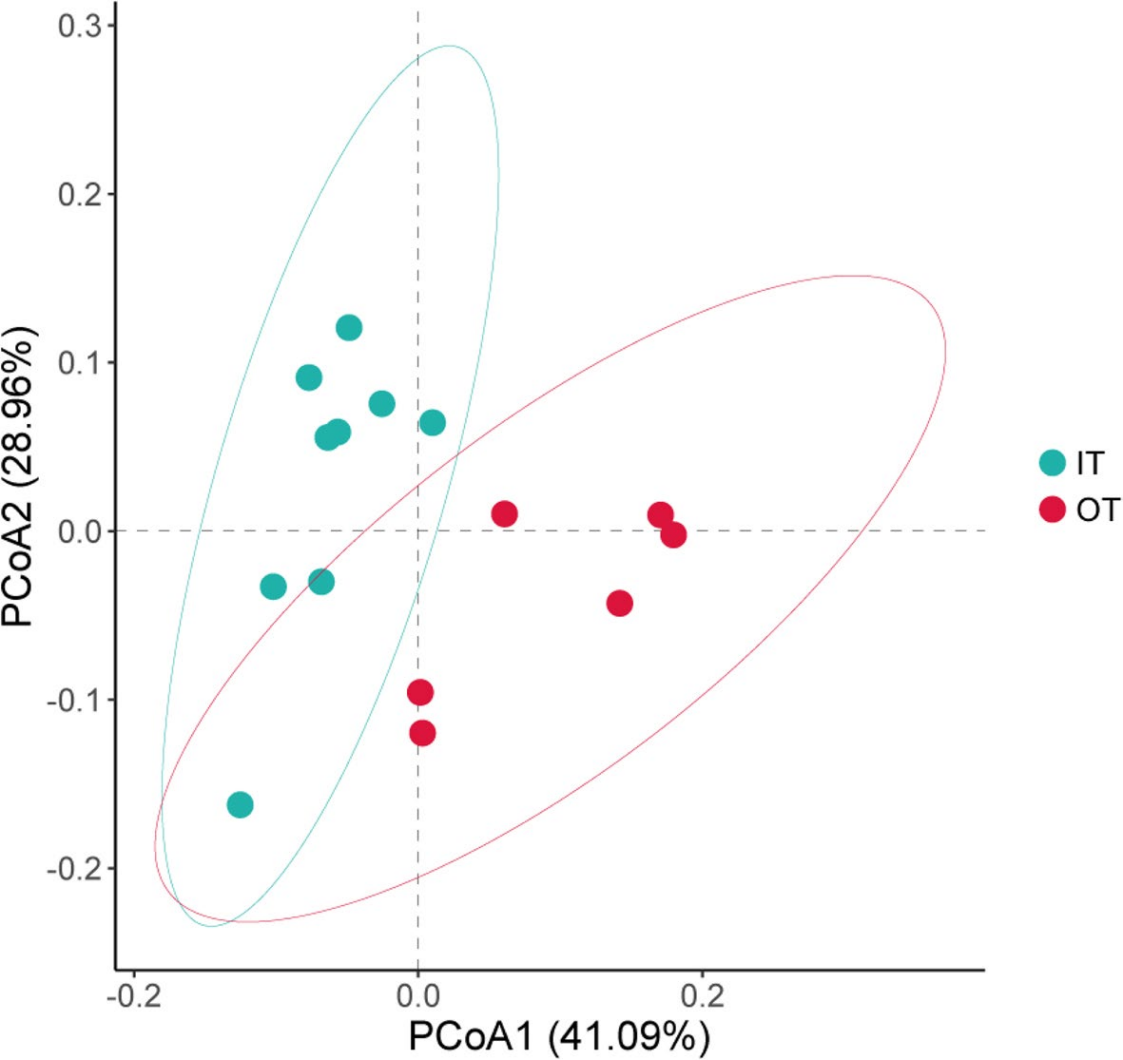
Principal coordinate analysis (PCoA) based on the unweighted UniFrac distance of different Shenxiantang tiankeng sites for the microbial communities

LEfSe analysis showed that the 131 microbial clades exhibited significant differences at different tiankeng sites (LDA score > 3.0). More specifically, there were 85 and 46 microbial clades at the IT and OT sites, respectively (Fig. S3). Most differential microbial clades were enriched in the IT. In particular, *Acidobacteria* (phyla), *Betaproteobacteria* (class), Gemmatimonadetes (phyla) and *Gemmatimonadales (*order*)* were specific to the IT; *Actinobacteria* (phyla), *Thermoleophilia* (class), *Solirubrobacterales* (order) and *Bradyrhizobium* (family) were specific to the OT. Principal coordinate analysis (PCoA) further visualized and explored the microbial community structure. The PCoA results showed that the microbial communities in the IT and OT had partial differentiation effects; the first two coordinate axes can explained 41.09 and 28.96% of the variation, respectively (Fig. 2).

### Metabolic pathways and function annotations

**Fig. 3:**
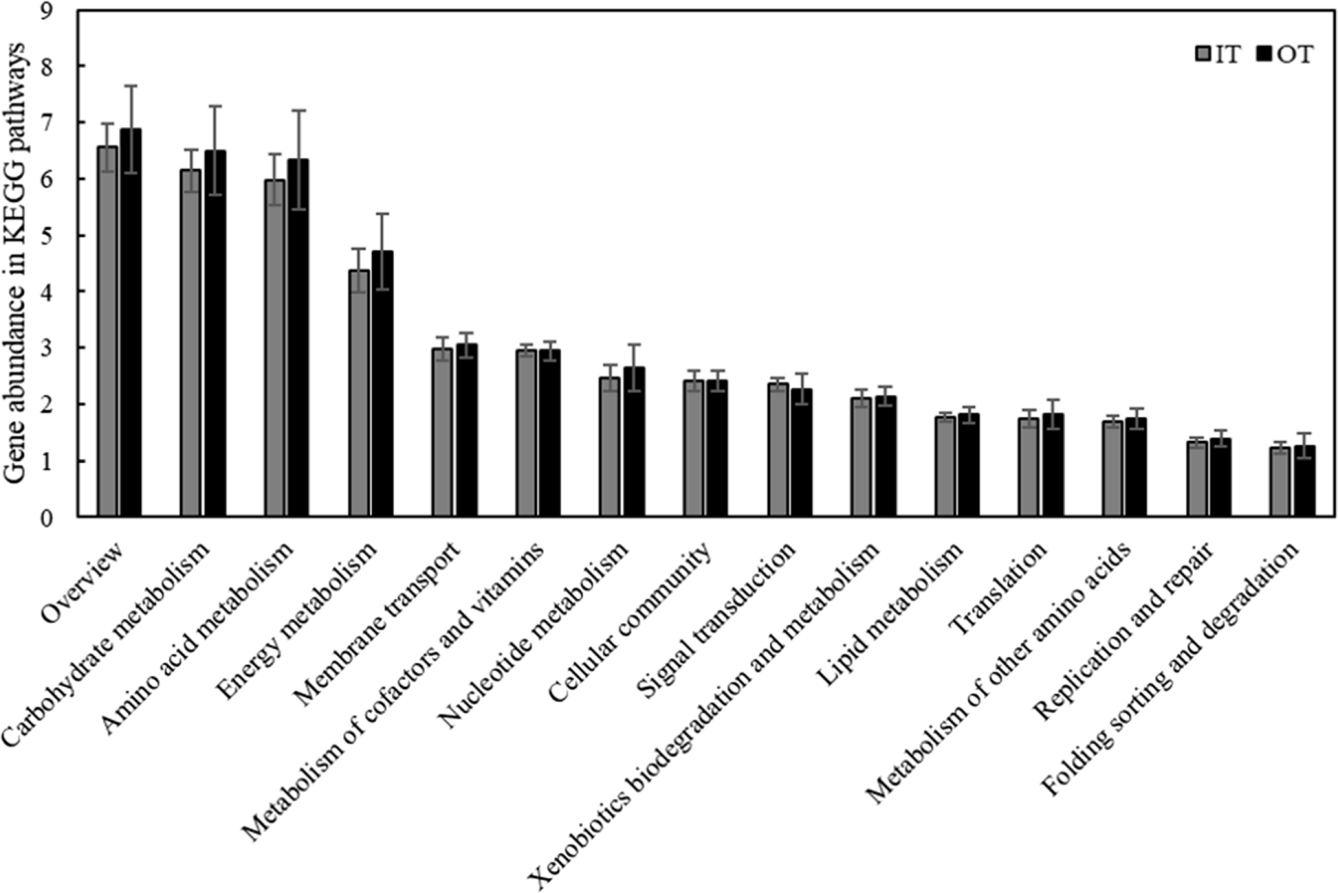
The abundance of genes associated with KEGG pathways (level 2) in different Shenxiantang tiankeng sites

**Fig. 4:**
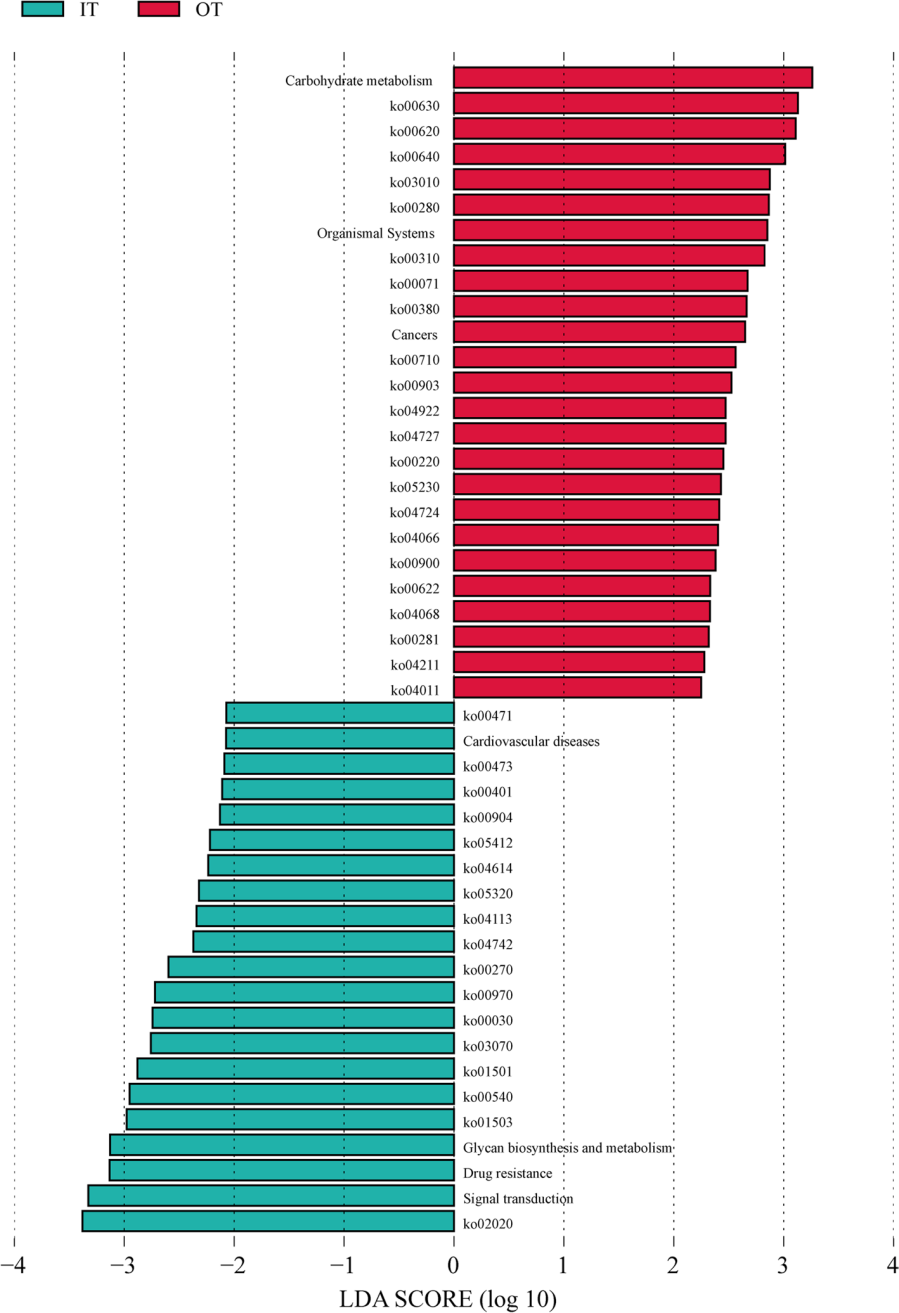
The histogram of LDA scores of level 3 functional categories with a threshold value of 2.0 in different Shenxiantang tiankeng sites. (Metabolism: ko00620, ko00630, ko00640, ko00280, ko00310, ko00071, ko00380, ko00710, ko00903, ko00220, ko00900, ko00622, ko00281, ko00471, ko00401, ko00904, ko00270, ko00030, ko00540; Genetic information processing: ko03010, ko00970; Organismal systems: ko04922, ko04727, ko04724, ko04211, ko04614, ko04742; Human diseases: ko05230, ko05412, ko05320, ko01501, ko01503; Environmental information processing: ko04066, ko04068, ko04011, ko03070, ko02020; Cellular processes: ko04113 [19])

KEGG pathway analysis contributed to the understanding of differences in potential microbial functions inside and outside the tiankeng. A total of 5060 KEGG orthologues (KO) were annotated at different sites, mainly belonging to metabolism, genetic information processing, environment information processing and cellular processes (Fig. S4). For level 2 KEGG orthologues, the top abundant functional pathways were carbohydrate metabolism, amino acid metabolism, energy metabolism and membrane transport. Among the top 15 functional pathways, 13 functional pathways exhibited higher levels at the OT site (Fig. 3). Furthermore, the LEfSe results showed the pathways with significant differences between the IT and OT sites. As shown in Fig. 4, IT sites were primarily associated with signal transduction, drug resistance, glycan biosynthesis and metabolism, membrane transport, carbohydrate metabolism, translation, amino acid metabolism, sensory system, cell growth and death, immune diseases, cardiovascular diseases, metabolism of terpenoids and polyketides, biosynthesis of other secondary metabolites and metabolism of other amino acids. However, the biomarkers of pathways at the OT sites were carbohydrate metabolism, translation, amino acid metabolism, organismal systems, lipid metabolism, cancers, energy metabolism, metabolism of terpenoids and polyketides, endocrine system, nervous system, signal transduction, metabolism of terpenoids and polyketides, xenobiotics biodegradation and metabolism and ageing.

**Fig. 5:**
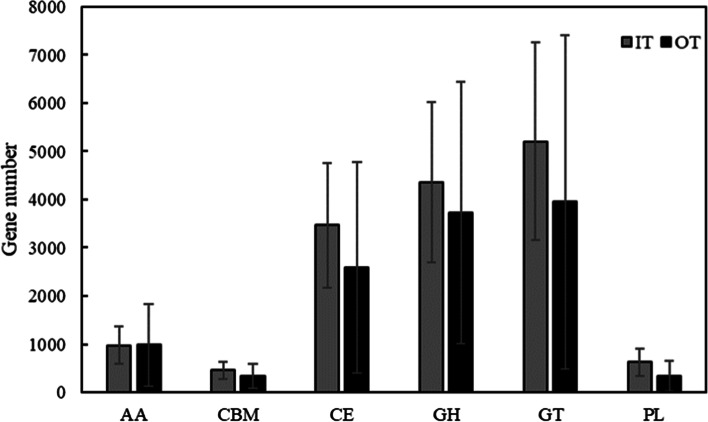
Variations of CAZy genes number in different Shenxiantang tiankeng sites. (AA: Auxiliary activities; CBM: Carbohydrate-binding modules, CE: Carbohydrate esterases; GH: Glycoside hydrolases; GT: Glycosyl transferases; PL: Polysaccharide lyases)

Based on the CAZy database, we further explored the characteristics of biomass conversion (CAZy genes) (Fig. 5). Genes related to the six distinct families were found in all soil samples. As shown in Fig. 5, almost all CAZy classes in the IT were higher than those in the OT, except for auxiliary activities (AA). In addition, some significant differences on CAZy families between the IT and OT sites were found (*P* < 0.05) (Fig. S5). The difference CAZy families were mainly belongs to glycoside hydrolases (GH) and glycosyl transferases (GT).

### Effects of soil characteristics on microbial composition

**Fig. 6:**
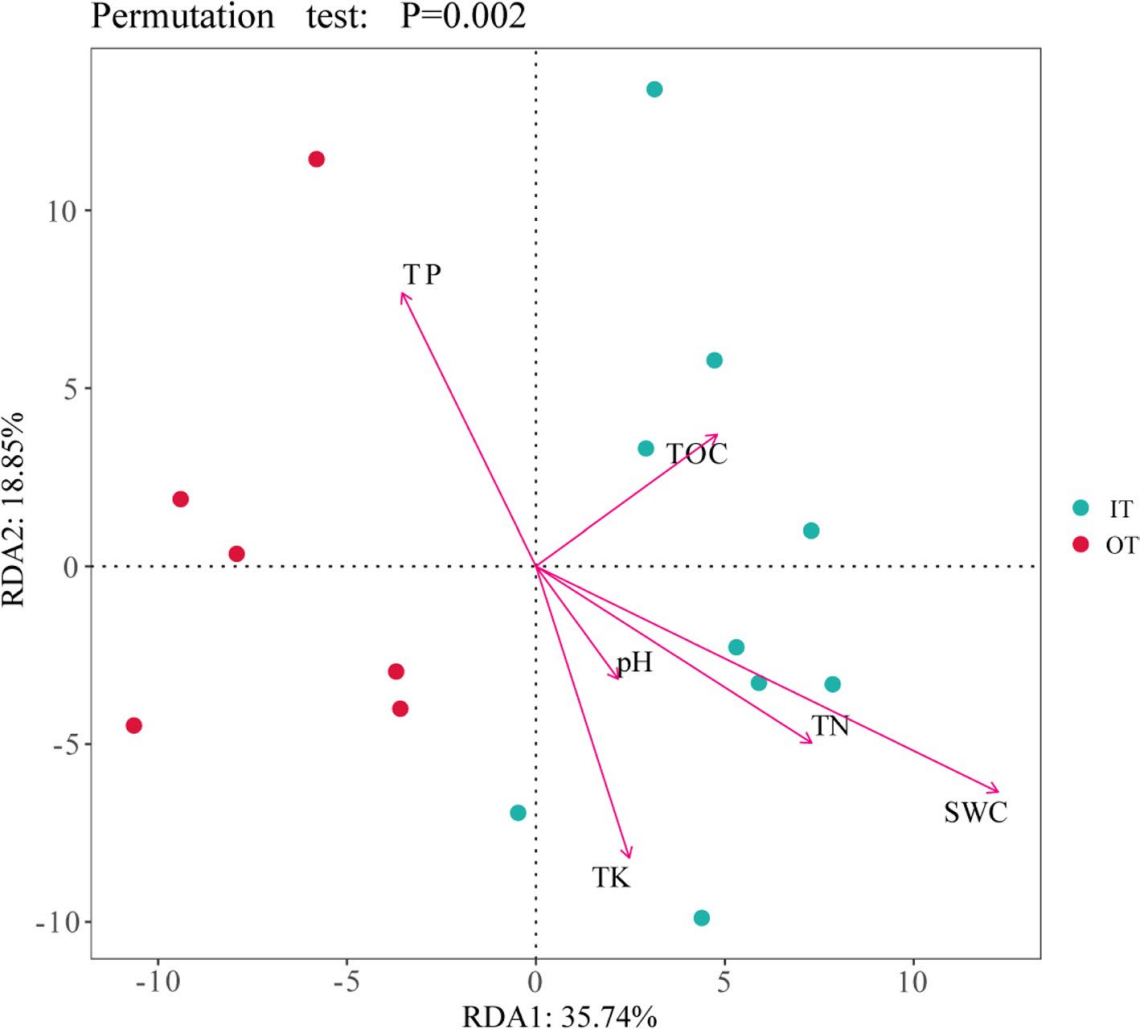
The redundancy analysis (RDA) of the soil microbial community with soil characteristics in different Shenxiantang tiankeng sites

RDA was applied to reveal the correlations between the soil characteristics and microbial community. The RDA axes explained 54.59% of the variations in the microbial communities. SWC and TN were the most key soil characteristics influencing the microbial communities (Fig. 6). Moreover, there were significant correlations based on the Spearman correlation coefficients between soil characteristics and the significantly changed microbial taxa at the phylum level (Fig. S6) (Table S3). The correlation analysis results showed a higher correlation coefficient between the significantly changed microbial taxa regarding SWC and TN than other soil characteristics at the karst tiankeng sites. TOC was significantly negatively correlated with *Actinobacteria* (*P* < 0.01). TK, TP and pH showed obtuse relationships with microbial taxa.

## Discussion

### Tiankeng habitat heterogeneity change the microbial communities diversity and composition

Due to the intensification of geological and climatic processes and human activities, karst regions have undergone severe environmental degradation and biodiversity loss [20–22]. Biodiversity conservation is a major challenge in degraded karst areas. As a unique habitat in karst ecosystems, karst tiankengs are highly heterogeneous. The heterogeneous properties of habitats can cause changes in biodiversity patterns [23]. In our study, the microbial community structure was significantly different between the inside and outside (Fig. 2). The microbial community diversity inside the tiankeng was significantly higher than that outside the tiankeng (Fig. S1). In addition, our studies found that karst tiankeng ecosystems have a stable microbial community composition. At the phylum and genus levels, the composition of the dominant populations was similar (Fig. 1). Although the microbial composition was similar, there were significant differences in relative abundance. Soil microbial communities can respond to changes in the soil environment by regulating the abundance of specific populations [24]. *Proteobacteria* was the most abundant phylum in both soil samples. These results were consistent with previous studies [25, 26]. *Actinobacteria*, *Chloroflexi* and *Acidobacteria* were also enriched in all soil samples. *Proteobacteria* are believed to be common in soil environments and have strong functional involvement in energy metabolism and carbon and nitrogen cycling [27]. Studies have shown that *Actinobacteria* prefer moist and cool environments [28, 29]. However, our study found that the abundance of *Actinobacteria* at the IT sites was lower. *Chloroflexi* and *Acidobacteria* are essential for the decomposition of organic matter and nutrient cycles [30]. This phenomenon may indicate that *Proteobacteria, Actinobacteria*, *Chloroflexi* and *Acidobacteria* were adapted to the habitat conditions in the karst tiankeng. Furthermore, LEfSe analysis results showed that IT sites were primarily associated with *Acidobacteria*, *Gemmatimonadetes* and *Chloroflexi*. However, the OT site biomarker was *Actinobacteria.* The different biomarkers may reflect that microorganisms with special functions at different sites can survive better. In general, the different habitat inside and outside the tiankeng affecting the diversity and composition of soil microbial community.

### Altered the function of tiankeng microbial community

Changes in microbial community composition and structure are often related to variations in microbial community function [31]. With metagenomic data, the functional capacities of microbiomes can be effectively analysed. In our study, the microbes participated in diverse pathways (Fig. S4). Most microorganisms are involved in metabolism, environmental information processing and cellular processes, which are of great significance for maintaining the basic metabolic activities of soil microorganisms [32]. Soil microbes coexist through a variety of metabolic processes and participate in a variety of complex nutrient circulation and energy flow processes in the karst tiankeng ecosystem. The distribution of pathways outside the tiankeng sitesshowed higher genes related to metabolism, particularly carbohydrate metabolism, amino acid metabolism and energy metabolism. The LEfSe analysis results also confirmed that the biomarker pathways of the OT sites mainly belonged to carbohydrate metabolism and amino acid metabolism. These results suggest that functional microbes involved in different dynamic activities may undergo dramatic changes in karst tiankeng ecosystems. The theory “nutrient limitation theory” proposed by Cherif and Loreau can better explain this phenomenon [33]. The poor soil nutrients outside the tiankeng make soil microbe growth more susceptible to restrictions from soil nutrient conditions. Therefore, soil microbes maintain community construction by changing survival strategies; that is, increasing the abundance of metabolic pathways. Compared with those outside the tiankeng sites, the rich soil nutrient conditions inside the tiankeng sites may make the microbes no longer limited by the soil nutrient resources. Microorganisms can easily obtain nutrients, which leads to a decrease in the abundance of metabolic pathways. This also reflects that the nutrient status of the interior of the tiankeng tends to be stable. In addition, the biomarker pathways at the IT and OT sites were also involved in human diseases, including drug resistance, immune diseases, cardiovascular diseases and cancers. Some studies have shown that exposure to microbial diversity can reduce certain diseases [34]. Therefore, attention should also be given to human diseases that may be caused by the loss of biodiversity in karst tiankeng ecosystems.

Microbial communities play an important role in terrestrial biogeochemistry and ecosystem C cycle [35]. CAZy genes represent a key C cycle function necessary in soil [36]. The abundance of CAZy class at the IT sites was higher than that at the OT sites, and the different CAZy families were mainly belonged to GH and GT. GH is related to the decomposition of lignin and humus [37]. The CAZy analysis indicated that most CAZy genes were severely inhibited in outside the tiankeng sites. The high abundance of GH genes indicates that the soil microorganisms at the IT sites have a strong potential for biomass decomposition. Rich vegetation at IT sites can produce a large amount of litter, which promotes the abundance of CAZy genes and ultimately improves the nutrient status of the soil in the tiankeng. Soil temperature is also an important factor affecting enzyme activity, and the wet and cold environment inside the tiankeng may also affect the activity of carbohydrate-active enzymes [38]. Microbial community metabolic pathway analysis indicated that the availabilities of resources and microbial interactions are important factors in the response of microbial communities to environmental change [39, 40]. Habitat differences inside and outside the tiankeng affect the participation of microbes in multiple survival strategies.

### Effect of soil environmental variables on tiankeng microbial community

Previous studies have reported that soil microbial communities have a close relationship with environmental variables and are characterized by heterogeneity at small spatial scales [41]. In fact, the closed habitat of karst tiankengs creates differences in these environmental variablesinside and outside the tiankeng. In this study, the soil physiochemical properties (except TP content) and vegetation characteristics (plant cover and Shannon–Wiener diversity) were higher inside the tiankeng (Table 1, S1). Rich soil nutrients can increase the diversity of the soil microbial community [42]. Similarly, we found higher microbial community diversity inside the tiankeng with more favourable soil conditions. RDA demonstrated that SWC and TN were the major drivers of the soil microbial community composition (Fig. 6). Changes in soil moisture conditions can influence the availability of water for the microbial community and can affect enzyme activities [43]. Many studies have confirmed that soil water content is a crucial factor in controlling the microbial community structure and diversity [1]. Within tiankengs, it is stable microclimates or moist habitats may be more conducive to the survival of the microbial community. In addition, TN has an effect on the soil microbial community composition. In fact, it has been reported that soil TN might affect a large number of microbial species in the soil [44]. Previous studies have showed that pH can substantially affect microbial communities [45]. However, our results showed that the impact of pH in the tiankeng was not significant, possibly because the pH values at different sites in the tiankeng did not vary greatly. Liu et al. (2012) showed that the microbial communities were also influenced by TP content [46]. However, TP is rather limited in the karst region and may have a minor effect on microbes. Taken together, the rich soil nutrient conditions may be one of the important drivers of the formation and evolution of the soil microbial community in karst tiankengs.

## Conclusion

Our results revealed the taxonomic and functional characteristics of the soil microbial communities in karst tiankengs. Compared with the area outside of the tiankeng, there were rich soil nutrients, vegetation Shannon–Wiener diversity and coverage inside the tiankeng. The soil microbial community composition (relative abundance of dominant phyla and genus) and structure were different inside and outside the tiankeng, and there was a higher microbial community diversity inside the tiankeng. A Venn diagram revealed 16,472 taxa of microbial communities, and the number of unique taxa was higher at the IT sites. Functional prediction analysis revealed that microbial communities were mainly involved in carbohydrate metabolism and amino acid metabolism. The biomarker pathways involved in human disease were identified in the karst tiankeng. Moreover, SWC and TN can affect the composition and structure of the microbial communities in karst tiankengs. Karst tiankengs are relatively independent special ecosystems, and our research may greatly expand our understanding of karst tiankeng systems. To understand better the detailed response of soil microorganisms to the unique habitat of karst tiankengs, and to evaluate the sustainability of karst tiankeng ecosystems, further studies should make full use of multiple omics techniques (e.g., metatranscriptomics).

## Materials and methods

### Field sites

**Fig. 7:**
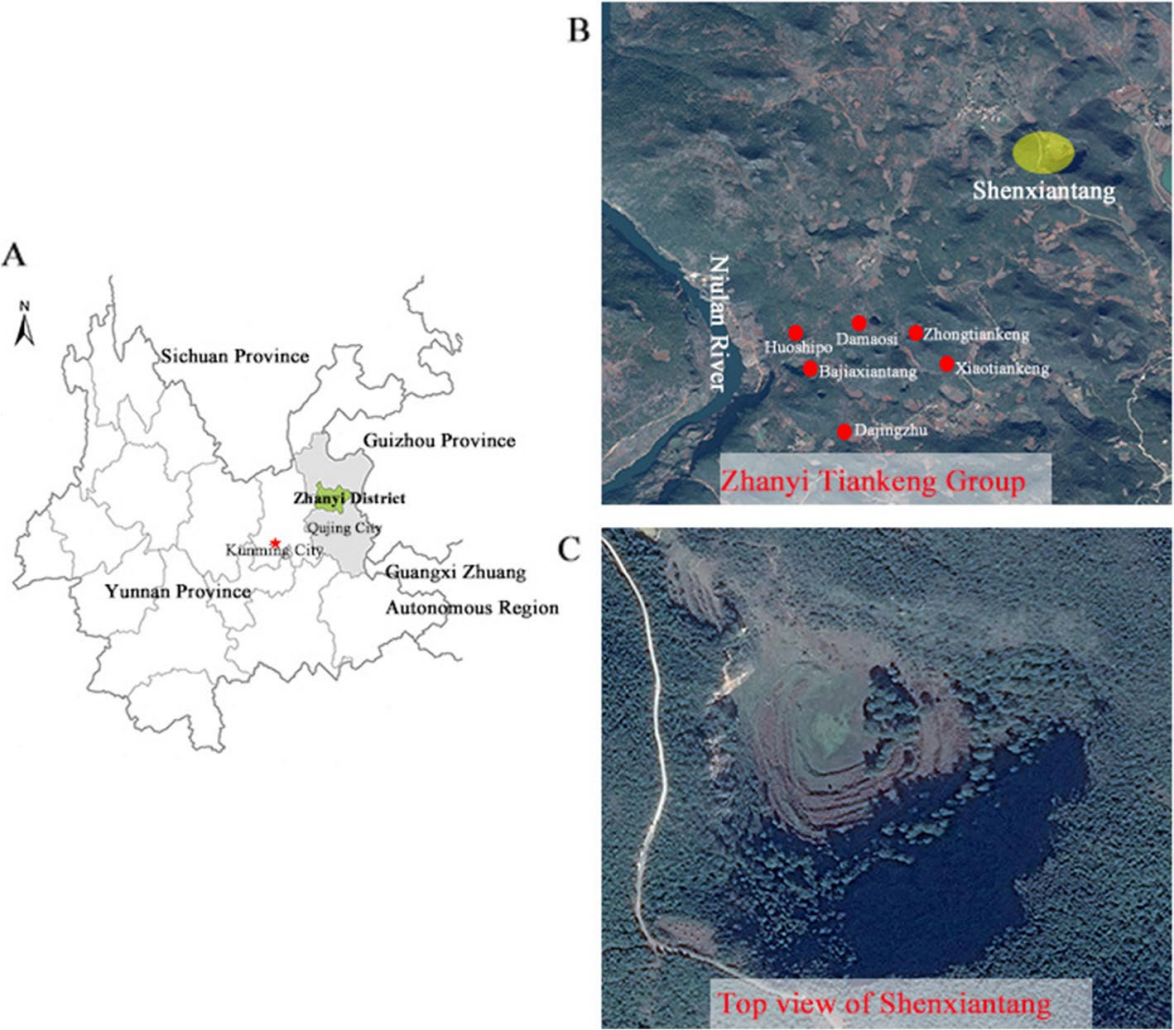
Geographical location of Shenxiantang tiankeng

The study area, “Shengxiantang” tiankeng (25°35′-25°57′N, 103°29′-103°39′E), is located in theHaifeng Natural Reserve, Yunnan Province, China (Fig. 7). The study area is characterized by the Fenglin karsts. This region has a typical subtropical plateau monsoon climate, with an average annual temperature of 14 °C, an annual precipitation of 1073.5–1089.7 mm and a relative humidity of 71%. The soil type is Yunnan red soil. The Shenxiantang tiankeng is 421.9 m long, 348.7 m wide and 148.7 m deep. A grassland plant community is dominant at the bottom of the tiankeng. Soil conditions and vegetation coverage were better in the eastern-southwestern slope, with almost no vegetation except for the nondegraded vertical wall in the western slope. The dominant trees were *Quercus pannosa* Hand.-Mazz., *Populus yunnanensis* Dode, *Broussonetia papyrifera*, and *Cyclobalanopsis glauca* (Thunb.) Oerst.

### Experimental design, plant investigation and soil sampling

We carried out this study in July 2019. The main vegetation in the karst tiankeng was evergreen broad-leaved forest and temperate coniferous forest. The typical sampling sites inside the tiankeng (IT) and outside the tiankeng (OT) were selected. The inside the Shenxiantang tiankeng included three sampling sites (each 20 × 20 m^2^) and the inside of the tiankeng included three different sampling sites (each 20 × 20 m^2^), i.e., sunny slope, shady slope and tiankeng bottom. The outside the tiankeng included two sampling sites, and located east and south of Shenxiantang tiankeng. Each sampling site contained 3 selected quadrats (1 × 1 m) along the diagonal. The plant coverage and species of each sampling site were determined, and the Shannon–Wiener diversity index was calculated. Soil samples were collected according to an “S” shape, and five points (upper 10 cm) of soil were mixed after the large pieces of debris were removed, homogenized and sieved (2 mm sieve). Thus, nine and six composite soil samples of the IT and OT sites were obtained, respectively. After being transported to the laboratory, each soil sample was divided into two parts. One part was placed at − 80 °C for molecular analysis. One part was placed at 4 °C to measure the soil physicochemical properties [47].

### Metagenomic sequencing

The microbial DNA was extracted from the soil by an E.Z.N.A.@ Soil DNA Kit (OMEGA, Norcross, GA, U.S.). The DNA integrity was assessed by 1% agarose gel electrophoresis (200 V, 30 min). To construct the paired-end library, 500-bp DNA fragments were generated using a Covaris S220 (Covaris Inc., Woburn, MA, U.S.). Afterwards, a paired-end library was constructed using the NEB Next® UltraTM DNA Library Prep Kit (Illumina, San Diego, CA, USA). Sequencing was performed on an Illumina PE150 platform (Illumina Inc., San Diego, CA, USA) at Sangon Biotech Co., Ltd. (Shanghai, China).

### Bioinformatics

FastQC (0.11.2) was used to visually evaluate the sequencing data quality of the samples. Trimmomatic was used to remove low-quality reads (having N bases or with a quality value < 20 or connector sequence in reads or length < 50 bp) [48]. The stitching software IDBA_UD (1.1.2) based on De Bruijn Graph principle was used to splicing and assembly high-quality reads. According to overlap relationship between reads, contigs was obtained and the best Kmer assembly results were selected [49]. Prodigal was used to predict the contig of the open reading frames (ORFs), and ORFs (length ≥ 100 bp) were translated to protein sequences [50]. Clustering 95% sequence identity (90% coverage) of the whole predicted gene sequences catalog was performed using CD-HIT (version 4.6), and the longest genes of every cluster were selected to construct a nonredundant gene catalog [51]. Obtain the abundance of genes in the sample through Bowtie2 (version 2.1.0) and Samtools (version 0.1.18) [52]. The assembled unigenes were compared with the NCBI non-redundant protein sequence database for blastp homology, and the function annotation and species information were obtained using DIAMOND (version 0.8.20) [53]. GhostKOALA (version 1.0) was used to compare protein sequence with KEGG database (Kyoto Encyclopedia of Genes and Genomes) to obtain KO number and pathways information [54]. HMMER3 (version 3.1b1) was used to compare CAZy database (Carbohydrate-Active Enzymes) and get the carbohydrate enzymes annotation [55]. The sequence results were submitted to the SRA at NCBI under the accession number PRJNA640943.

### Statistical analysis

The microbial community Shannon-Wiener diversity index was calculated by Mothur (version v.1.30.1, http://www.mothur.org/wiki). ANOVA analyses of differences in the microbial Shannon diversity index and soil physicochemical properties. Based on weighted Unifrac results, principal coordinate analysis (PCoA) was performed in QIIME. The potential microbial taxa and functional biomarkers were analyzed by linear discriminant analysis effect size (LEfSe) (http://huttenhower.sph.harvard.edu/galaxy/root?tool_id=PICRUSt_normalize). The Mantel test of discern correlations between the soil physicochemical characteristics and microbial communities and was performed using the R vegan package. Redundancy analysis (RDA) was used to analyze the correlative relationships between microbial community and soil physiochemical properties by Canoco (version 5 for windows; Ithaca, NY, United States). Spearman correlation analysis was used to test for relationships between soil characteristics and significantly differed microbial taxa. The significance level was detected at the *P* < 0.05 and applied for all comparisons. The statistical analyses were performed using SPSS software (version 22.0).

## Supplementary Information

**Additional file 1: Table S1**. Plant characteristics of different Shenxiantang tiankeng sites.

**Additional file 2: Table S2**. The relative abundance of microbial community at the domain level of different Shenxiantang tiankeng sites.

**Additional file 3: Table S3**. The microbial taxa that showed significant different between the inside the tiankeng sites and outside the tiankeng sites at the phyla level based on ANOVA analysis.

**Additional file 4: Figure S1**. The microbial community Shannon-Wiener index of different Shenxiantang tiankeng sites.

**Additional file 5: Figure S2**. Venn diagram of species of different Shenxiantang tiankeng sites.

**Additional file 6: Figure S3**. The histogram of LDA scores of microbial clades with a threshold value of 3.0 in different Shenxiantang tiankeng sites.

**Additional file 7: Figure S4**. The abundance of KEGG pathway in different Shenxiantang tiankeng sites.

**Additional file 8: Figure S5**. Variations on CAZy gene in different Shenxiantang tiankeng sites. * indicates a significant correlation at *P* < 0.05.

**Additional file 9: Figure S6**. Correlations between soil characteristics and significantly differed microbial taxa. * indicates a significant correlation at *P* < 0.05, ** indicates a significant correlation at *P* < 0.01.

## Data Availability

The raw data of metagenomic analysis are available from the Sequence Read Archive (SRA) database of National Center for Biotechnology Information (NCBI) official website (accession number: PRJNA640943).
