# Supplementary material for: Metagenomic analysis reveals the different characteristics of microbial communities inside and outside the karst tiankeng
Source: BMC Microbiol. 2022 Apr 26;22:115. doi: 10.1186/s12866-022-02513-1 (PMC9040234; doi:10.1186/s12866-022-02513-1)
Supplement: Supplementary file 1 — Additional file 1: Table S1. Plant characteristics of different Shenxiantang tiankeng sites. [file 12866_2022_2513_MOESM1_ESM.docx]

| **Site** | **Plant coverage (%)** | **Dominant Species** | **Shannon-Wiener index** |
| --- | --- | --- | --- |
| IT | 75 | *Myrsine africana* Linn.  *Debregeasia orientalis* C. J. Chen  *Swida oblonga*  *Cyclobalanopsis glauca*  *Keteleeria evelyniana* Mast. | 2.25 |
| OT | 62 | *Myrsine africana* Linn.  *Viburnum propinquum*  *Cyclobalanopsis glauca* | 1.97 |
