# Supplementary material for: Metagenomic analysis reveals the different characteristics of microbial communities inside and outside the karst tiankeng
Source: BMC Microbiol. 2022 Apr 26;22:115. doi: 10.1186/s12866-022-02513-1 (PMC9040234; doi:10.1186/s12866-022-02513-1)
Supplement: Supplementary file 2 — Additional file 2: Table S2. The relative abundance of microbial community at the domain level of different Shenxiantang tiankeng sites. [file 12866_2022_2513_MOESM2_ESM.docx]

| **Group** | **Bacteria** | **Archaea** | **Fungi** | **Viruses** |
| --- | --- | --- | --- | --- |
| IT | 98.91% | 0.75% | 0.30% | 0.03% |
| OT | 99.30% | 0.47% | 0.20% | 0.03% |
