# Supplementary material for: Metagenomic analysis reveals the different characteristics of microbial communities inside and outside the karst tiankeng
Source: BMC Microbiol. 2022 Apr 26;22:115. doi: 10.1186/s12866-022-02513-1 (PMC9040234; doi:10.1186/s12866-022-02513-1)
Supplement: Supplementary file 3 — Additional file 3: Table S3. The microbial taxa that showed significant different between the inside the tiankeng sites and outside the tiankeng sites at the phyla level based on ANOVA analysis. [file 12866_2022_2513_MOESM3_ESM.docx]

|  | **ID** | **IT** | **OT** | ***P*** |
| --- | --- | --- | --- | --- |
| Bacteria | p__Actinobacteria | 19.5271±4.5111 | 34.2360±9.4815 | ** |
|  | p__Gemmatimonadetes | 2.5241±0.6837 | 0.7883±0.2340 | ** |
|  | p__Bacteria_noname | 2.2932±0.3375 | 1.7724±0.2717 | ** |
|  | p__Cyanobacteria | 1.6475±0.3327 | 1.0415±0.1037 | ** |
|  | p__Planctomycetes | 1.4104±0.2186 | 1.0055±0.2157 | ** |
|  | p__candidate_division_NC10 | 0.4957±0.1222 | 0.2735±0.0711 | ** |
|  | p__candidate_division_Zixibacteria | 0.2663±0.0562 | 0.1058±0.0144 | ** |
|  | p__Candidatus_Latescibacteria | 0.2103±0.0781 | 0.0468±0.0183 | ** |
|  | p__Spirochaetes | 0.1329±0.0186 | 0.0848±0.0100 | ** |
|  | p__Chlorobi | 0.1242±0.0198 | 0.0729±0.0100 | ** |
|  | p__Candidatus_Dadabacteria | 0.1150±0.0204 | 0.0617±0.0175 | ** |
|  | p__Candidatus_Kryptonia | 0.0927±0.0167 | 0.0437±0.0059 | ** |
|  | p__Candidatus_Omnitrophica | 0.0652±0.0091 | 0.0345±0.0084 | ** |
|  | p__Aquificae | 0.0552±0.0058 | 0.0337±0.0048 | ** |
|  | p__Deferribacteres | 0.0481±0.0092 | 0.0232±0.0046 | ** |
|  | p__Thermodesulfobacteria | 0.0457±0.0057 | 0.0276±0.0045 | ** |
|  | p__Ignavibacteriae | 0.0385±0.0065 | 0.0184±0.0043 | ** |
|  | p__Nitrospinae | 0.0397±0.0073 | 0.0240±0.0062 | ** |
|  | p__Thermotogae | 0.0301±0.0045 | 0.0184±0.0023 | ** |
|  | p__candidate_division_WOR-3 | 0.0196±0.0046 | 0.0091±0.0015 | ** |
|  | p__Rhodothermaeota | 0.0157±0.0025 | 0.0066±0.0016 | ** |
|  | p__Candidatus_Uhrbacteria | 0.0115±0.0027 | 0.0071±0.0017 | ** |
|  | p__Candidatus_Amesbacteria | 0.0074±0.0015 | 0.0050±0.0008 | ** |
|  | p__Candidatus_Peregrinibacteria | 0.0097±0.0011 | 0.0068±0.0009 | ** |
|  | p__Candidatus_Marinimicrobia | 0.0094±0.0009 | 0.0058±0.0023 | ** |
|  | p__Candidatus_Giovannonibacteria | 0.0115±0.0029 | 0.0063±0.0014 | ** |
|  | p__Fibrobacteres | 0.0097±0.0010 | 0.0052±0.0020 | ** |
|  | p__candidate_division_Hyd24-12 | 0.0068±0.0021 | 0.0022±0.0006 | ** |
|  | p__Candidatus_Magasanikbacteria | 0.0083±0.0022 | 0.0049±0.0010 | ** |
|  | p__Dictyoglomi | 0.0089±0.0020 | 0.0052±0.0019 | ** |
|  | p__Candidatus_Yanofskybacteria | 0.0076±0.0010 | 0.0050±0.0010 | ** |
|  | p__Lentisphaerae | 0.0051±0.0013 | 0.0027±0.0011 | ** |
|  | p__Candidatus_Cloacimonetes | 0.0047±0.0012 | 0.0023±0.0008 | ** |
|  | p__Candidatus_Kaiserbacteria | 0.0049±0.0011 | 0.0032±0.0004 | ** |
|  | p__Candidatus_Nomurabacteria | 0.0054±0.0008 | 0.0037±0.0011 | ** |
|  | p__Chrysiogenetes | 0.0062±0.0012 | 0.0032±0.0006 | ** |
|  | p__Candidatus_Collierbacteria | 0.0025±0.0008 | 0.0013±0.0006 | ** |
|  | p__Elusimicrobia | 0.0030±0.0011 | 0.0013±0.0005 | ** |
|  | p__Candidatus_Wolfebacteria | 0.0027±0.0008 | 0.0014±0.0004 | ** |
| Archaea | p__Euryarchaeota | 0.3991±0.0729 | 0.2795±0.0717 | ** |
|  | p__Candidatus_Bathyarchaeota | 0.0460±0.0120 | 0.0286±0.0077 | ** |
|  | p__Archaea_noname | 0.0372±0.0046 | 0.0226±0.0058 | ** |
