# Supplementary figures and images for: Metagenomic analysis reveals the different characteristics of microbial communities inside and outside the karst tiankeng
Source: BMC Microbiol. 2022 Apr 26;22:115. doi: 10.1186/s12866-022-02513-1 (PMC9040234; doi:10.1186/s12866-022-02513-1)

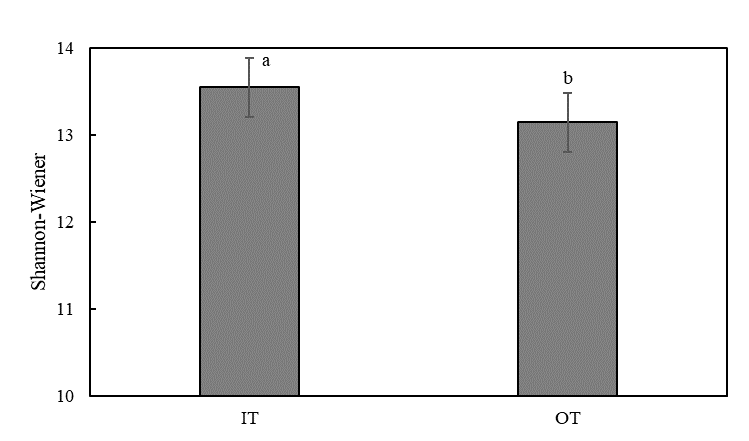

Supplement: Supplementary file 4 — Additional file 4: Figure S1. The microbial community Shannon-Wiener index of different Shenxiantang tiankeng sites. [file 12866_2022_2513_MOESM4_ESM.docx]

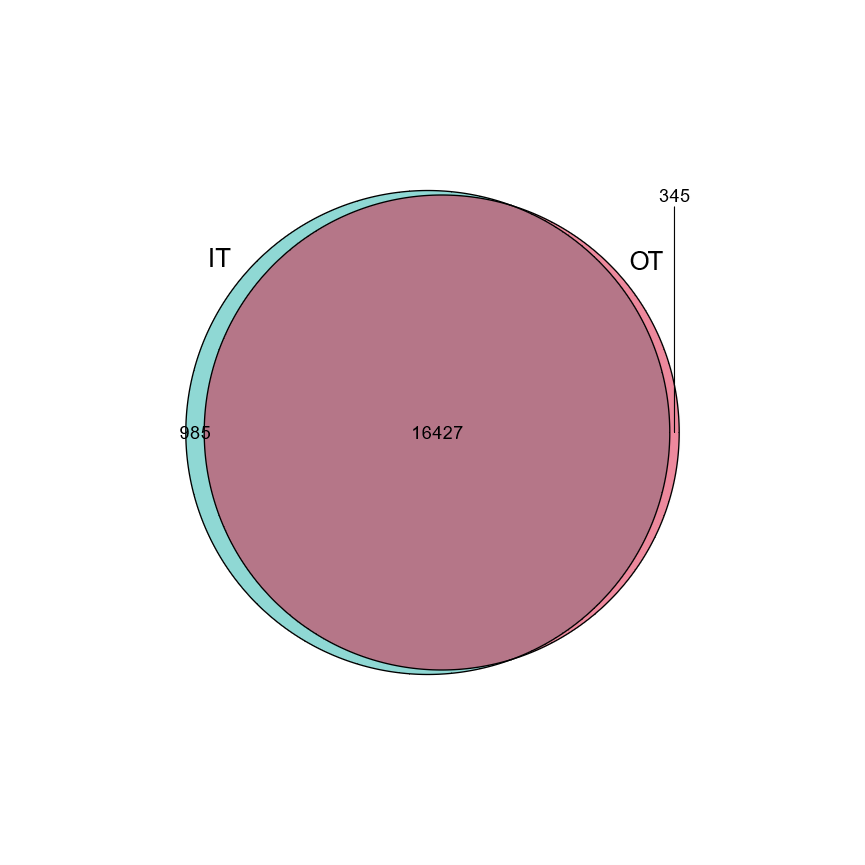

Supplement: Supplementary file 5 — Additional file 5: Figure S2. Venn diagram of species of different Shenxiantang tiankeng sites. [file 12866_2022_2513_MOESM5_ESM.docx]

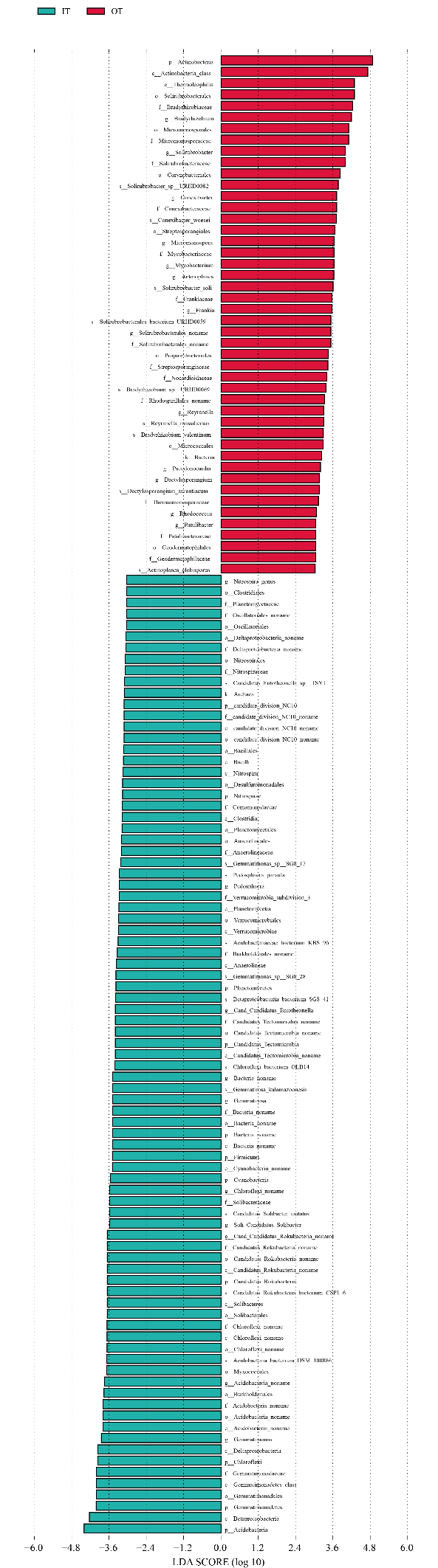

Supplement: Supplementary file 6 — Additional file 6: Figure S3. The histogram of LDA scores of microbial clades with a threshold value of 3.0 in different Shenxiantang tiankeng sites. [file 12866_2022_2513_MOESM6_ESM.docx]

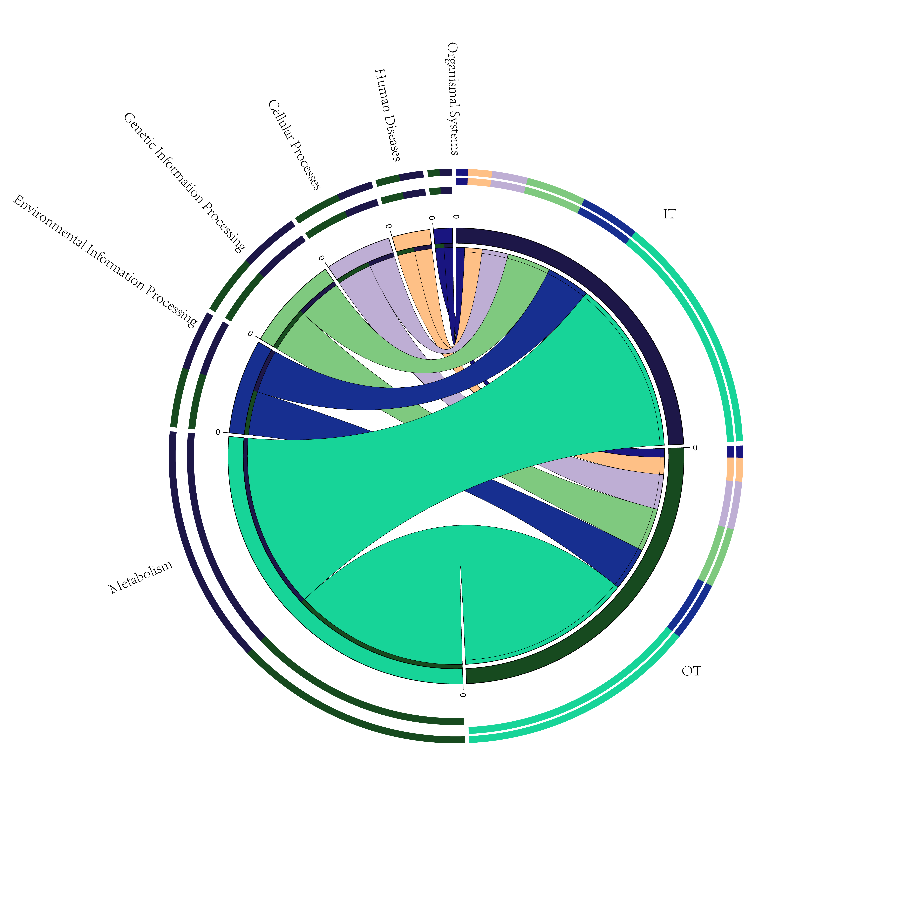

Supplement: Supplementary file 7 — Additional file 7: Figure S4. The abundance of KEGG pathway in different Shenxiantang tiankeng sites. [file 12866_2022_2513_MOESM7_ESM.docx]

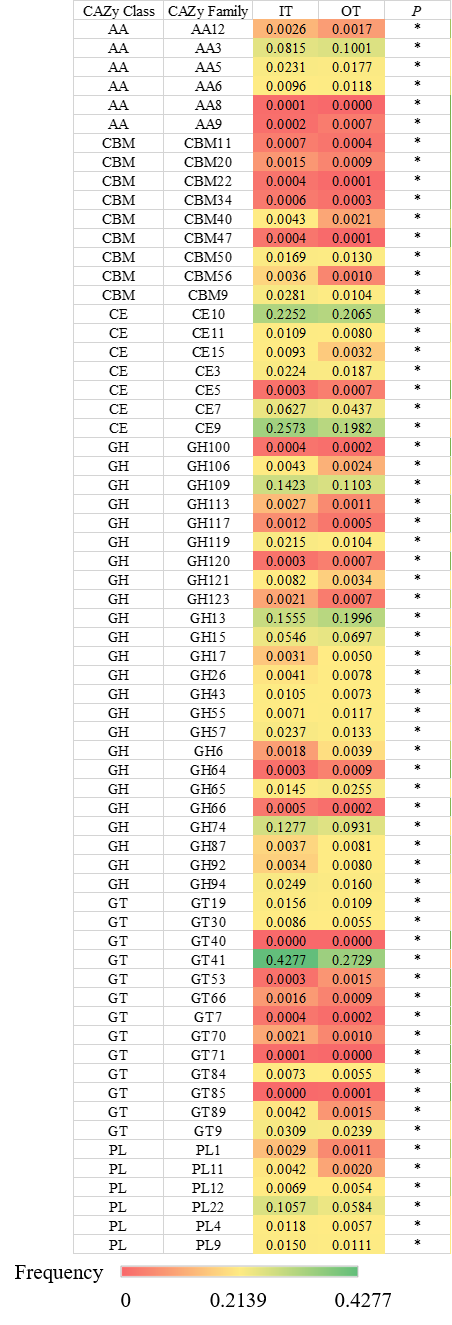

Supplement: Supplementary file 8 — Additional file 8: Figure S5. Variations on CAZy gene in different Shenxiantang tiankeng sites. * indicates a significant correlation at P < 0.05. [file 12866_2022_2513_MOESM8_ESM.docx]

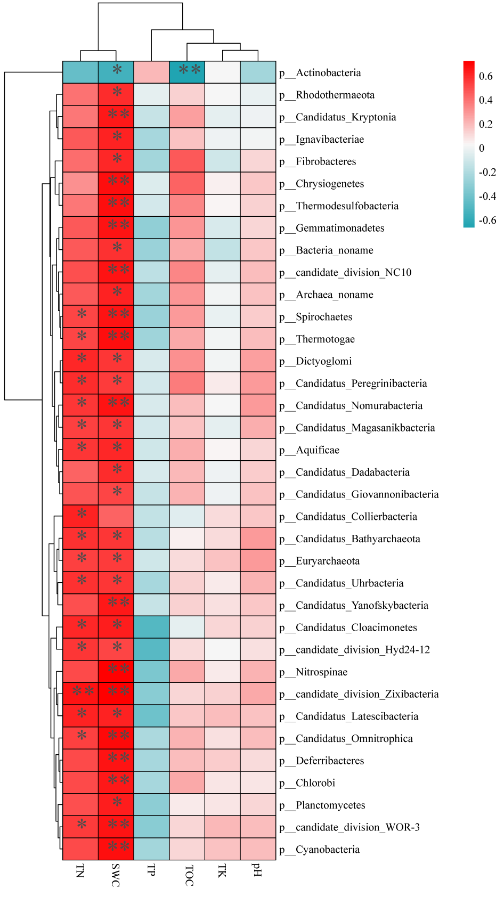

Supplement: Supplementary file 9 — Additional file 9: Figure S6. Correlations between soil characteristics and significantly differed microbial taxa. * indicates a significant correlation at P < 0.05, ** indicates a significant correlation at P < 0.01. [file 12866_2022_2513_MOESM9_ESM.docx]
